# A Comprehensive Approach to Sequence-oriented IsomiR annotation (CASMIR): demonstration with IsomiR profiling in colorectal neoplasia

**DOI:** 10.1186/s12864-018-4794-7

**Published:** 2018-05-25

**Authors:** Chung Wah Wu, Jared M. Evans, Shengbing Huang, Douglas W. Mahoney, Brian A. Dukek, William R. Taylor, Tracy C. Yab, Thomas C. Smyrk, Jin Jen, John B. Kisiel, David A. Ahlquist

**Affiliations:** 1 Division of Gastroenterology and Hepatology, Mayo Clinic, 200 First Street SW, Rochester, MN 55905 USA; 2 Division of Biomedical Statistics and Informatics, Mayo Clinic, Rochester, MN USA; 3 Division of Bioinformatics and Computational Biology, University of Minnesota Rochester, Rochester, MN USA; 4 Division of Anatomic Pathology, Mayo Clinic, Rochester, MN USA; 5 Genome Analysis Core, Medical Genome Facility, Mayo Clinic, Rochester, MN USA; 6 Division of Experimental Pathology, Mayo Clinic, Rochester, MN USA

**Keywords:** MicroRNAs, High-throughput nucleotide sequencing, Colorectal neoplasms, Gene expression profiling, Biomarkers

## Abstract

**Background:**

MicroRNA (miRNA) profiling is an important step in studying biological associations and identifying marker candidates. miRNA exists in isoforms, called isomiRs, which may exhibit distinct properties. With conventional profiling methods, limitations in assay and analysis platforms may compromise isomiR interrogation.

**Results:**

We introduce a comprehensive approach to sequence-oriented isomiR annotation (CASMIR) to allow unbiased identification of global isomiRs from small RNA sequencing data. In this approach, small RNA reads are maintained as independent sequences instead of being summarized under miRNA names. IsomiR features are identified through step-wise local alignment against canonical forms and precursor sequences. Through customizing the reference database, CASMIR is applicable to isomiR annotation across species. To demonstrate its application, we investigated isomiR profiles in normal and neoplastic human colorectal epithelia. We also ran miRDeep2, a popular miRNA analysis algorithm to validate isomiRs annotated by CASMIR. With CASMIR, specific and biologically relevant isomiR patterns could be identified. We note that specific isomiRs are often more abundant than their canonical forms. We identify isomiRs that are commonly up-regulated in both colorectal cancer and advanced adenoma, and illustrate advantages in targeting isomiRs as potential biomarkers over canonical forms.

**Conclusions:**

Studying miRNAs at the isomiR level could reveal new insight into miRNA biology and inform assay design for specific isomiRs. CASMIR facilitates comprehensive annotation of isomiR features in small RNA sequencing data for isomiR profiling and differential expression analysis.

**Electronic supplementary material:**

The online version of this article (10.1186/s12864-018-4794-7) contains supplementary material, which is available to authorized users.

## Background

MicroRNAs (miRNAs) are crucial regulators of gene expression in plants and animals. Conventionally, a list curated by the miRNA database (miRBase.org) defines the unique sequences referred to as the canonical forms. However, accumulating evidence from deep sequencing suggests that miRNAs are heterogeneous in length and sequence, and the full array of isoforms is referred to as isomiRs [1].

Mechanisms governing the generation of isomiRs are not completely understood. For context, biogenesis of miRNA typically involves nuclear cleavage of primary miRNA into precursor miRNA, and cytoplasmic cleavage of pre-miRNA by Dicer into miRNA duplex [2]. The final products are 17–25 nucleotides in length, called mature miRNAs. Variants could originate from imprecise cleavage by Drosha or Dicer, generating sequences of different lengths [3]. Alternatively, isomiRs could arise from post-transcriptional modifications initiated by nucleotidyl transferase, which predominantly adds specific nucleotides to pre-miRNA or mature miRNA ends [4–6]. IsomiRs may vary from their canonical forms in both abundance and stability, and affect different downstream pathways [7, 8]. For detection, the widely used predesigned assays intended to detect canonical forms may variably quantify isomiRs.

Profiling miRNA at the isomiR level has been challenging for a few reasons. Hybridization based approaches such as northern blot, microarray, and PCR array are inefficient in differentiating highly similar sequences, limited in throughput, and require prior knowledge of the variant sequences. Although next generation sequencing (NGS) can interrogate sequences at single nucleotide resolution, current bioinformatic workflows typically summarize reads under miRNA names uninformative of variants. Several in silico tools support isomiR identification. However, among them there is a lack of consensus on isomiR classification. Methods based on sequence complementarity to precursor [9, 10], location of modified nucleotides [11, 12], pattern of modified nucleotides [13], or pre-defined isomiRs [14, 15] have been described depending on research interest or context, leading to incomplete isomiR identification and a lack of comparability. In this study, we take a unique approach by maintaining miRNA reads as independent sequences to preserve variants. Sequences are tested for comprehensive isomiR features through stepwise local alignments against canonical miRNA sequences and precursor sequences. Without relying on pre-defined isomiR sequences, this approach would thus allow unbiased and comprehensive identification of isomiRs.

As a test set of this comprehensive approach to sequence-oriented isomiR annotation (CASMIR) workflow, we demonstrate its application in identifying differentially expressed isomiR in colorectal neoplasia. Colorectal cancer (CRC) is the third most common cancer and the second most common overall cause of cancer death in the U.S. [16]. Numerous studies have investigated miRNA profiles in association with CRC via different assay platforms [17–19]; however, none has interrogated miRNA variants. Herein, we describe small RNA sequencing on 20 normal, 26 advanced adenoma and 35 CRC formalin-fixed paraffin-embedded (FFPE) tissue samples. We annotated global isomiR and identified differentially expressed isomiRs between disease and control groups. Last, as a proof-of-concept, we evaluated potential advantages of targeting isomiRs as CRC biomarkers, and conduct validation using quantitative reverse-transcription PCR (qRT-PCR) in an independent patient cohort including 30 normal, 20 advanced adenoma and 40 CRCs.

## Methods

### Comprehensive isomiR classification

**Fig. 1:**
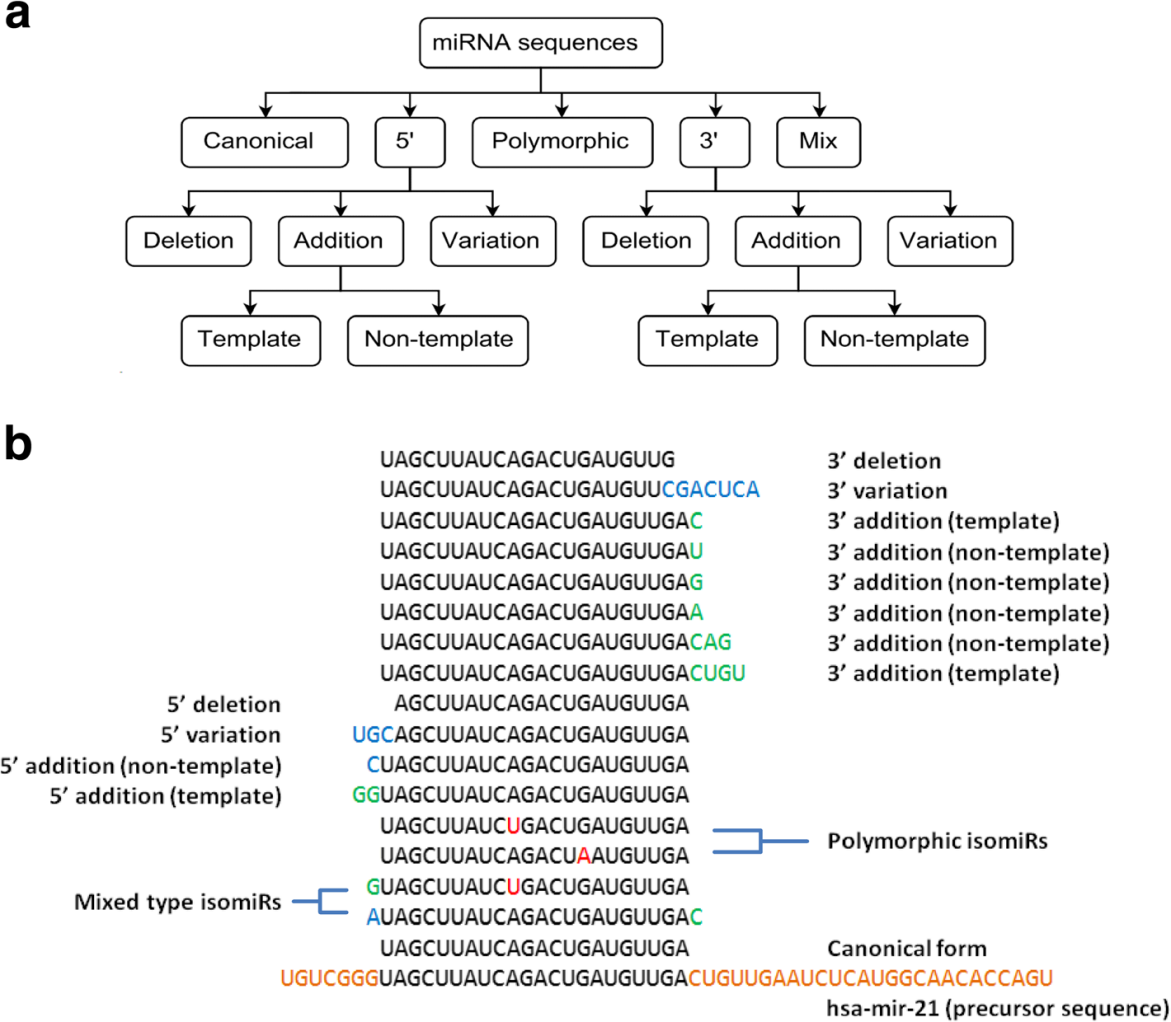
**a** Comprehensive isomiR classification. **b** illustration of different types of isomiR using hsa-miR-21-5p as an example

To facilitate isomiR annotation, we introduced a novel classification to encompass all possible forms of variants (Fig. 1a and b). miRNA sequences are categorized into five mutually exclusive classes: 1) Canonical forms, which are reference mature miRNA sequences defined in miRNA database; 2) 5′ isomiRs, sequences with changes at 5′ end with respect to the canonical form; 3) 3′ isomiRs, sequences with changes at 3′ end; 4) polymorphic isomiRs, sequences with changes in-between the first and last nucleotides; 5) mixed type isomiRs, sequences with at least two of 5′, 3′, and polymorphic isomiR features. Five prime and 3′-isomiRs are further classified into three mutually exclusive classes: deletion, addition and variation forms. Deletion refers to nucleotide(s) loss at 5′ or 3′ ends. Addition refers to nucleotide(s) addition at 5′ or 3′ ends. Variation refers to non-template changes of 5′ or 3′ ends’ nucleotides. Variation could extend the original length with additional nucleotides. Addition isomiRs can be further classified into template or non-template forms, which refers to whether the additional nucleotides can be aligned to precursor sequences. In cases of mature miRNA with more than one precursor, if the additional nucleotide(s) can align with any of the precursors, the isomiR is assigned template form. If the additional nucleotide(s) do not match any of the precursors, the isomiR is defined non-template form. It is possible that addition isomiRs matching their precursor sequences could acquire the additional base(s) through non-template pathways; however, it is not possible to distinguish the true origin of the additional bases by bioinformatics analysis. Non-template isomiRs are likely generated primarily by modifications mediated via nucleotidyl transferase.

### Test sample sets and patient characteristics

Two independent sets of colorectal tissue samples were investigated. *Discovery set* included 20 normal colon, 26 advanced adenoma, and 35 CRC tissues. *Validation set* included 30 normal colon, 20 advanced adenoma, and 40 CRC tissues (Table 1). All tissues were formalin-fixed-paraffin embedded (FFPE) samples obtained from Mayo Clinic archives on patients seen between Sep 2007 and Aug 2013, and were reviewed by an expert gastrointestinal pathologist. All cancer and advanced adenoma tissues contain above 80% neoplastic cells. An effort was made to balance age and gender within each set. Patients with a family history of familial adenomatous polyposis or hereditary non-polyposis, previous colorectal surgery, or neo-adjuvant therapy were excluded. The study was approved by the Mayo Clinic Institutional Review Board.

**Table 1:** Patient Characteristics: *discovery and validation sets* for isomiR profiling

	Discovery set			Validation set		
	Normal Colon	Advanced Adenoma^b^	CRC	Normal Colon	Advanced Adenoma^b^	CRC
No. of cases	20	26	35	30	20	40
Age						
Mean ± SD	62 ± 5	62 ± 11	59 ± 10	61 ± 13	66 ± 11	70 ± 13
Gender, number (%)						
Female	9 (45)	13 (50)	17 (49)	15 (50)	6 (30)	22 (55)
Location, number (%)						
Proximal^a^		13 (50)	17 (49)		13 (65)	17 (43)
TNM stage, number (%)						
I			6 (17)			1 (3)
II			10 (29)			12 (30)
III			15 (43)			26 (65)
IV			4 (11)			1 (3)

^a^Proximal lesions include tumors at or proximal to the splenic flexure, and distal lesions are those distal to the splenic flexure

^b^Advanced adenoma includes 1) adenoma measuring ≥1 cm in the greatest dimension, with high-grade dysplasia, or with ≥25% villous histologic features, and 2) Sessile serrated adenoma ≥ 1 cm

### Small RNA sequencing

Small RNA sequencing was performed using the *discovery set*. Total RNA was extracted using RecoverAll Total Nucleic Acid Isolation Kit for FFPE (Thermo Fisher Scientific, Massachusetts, USA). A total of 500 nano-grams RNA per sample were used to prepare small RNA libraries using NEBNext Small RNA Library Prep Set following manufacturer’s guide (New England Biolabs, Massachusetts, USA). Number of cycles for PCR enrichment was adjusted based on initial library amount quantified by qPCR. Up to 24 different libraries were indexed and pooled in a single sequencing lane with randomization. The libraries were sequenced at 50 bp single-read mode on an Illumina HiSeq 2000 sequencing system (Illumina, California, USA). However, this workflow does not rely on sequencer-specific code and is compatible with other common sequencing platforms.

### Implementation of CASMIR

#### Trimming, normalization and size filtering

**Fig. 2:**
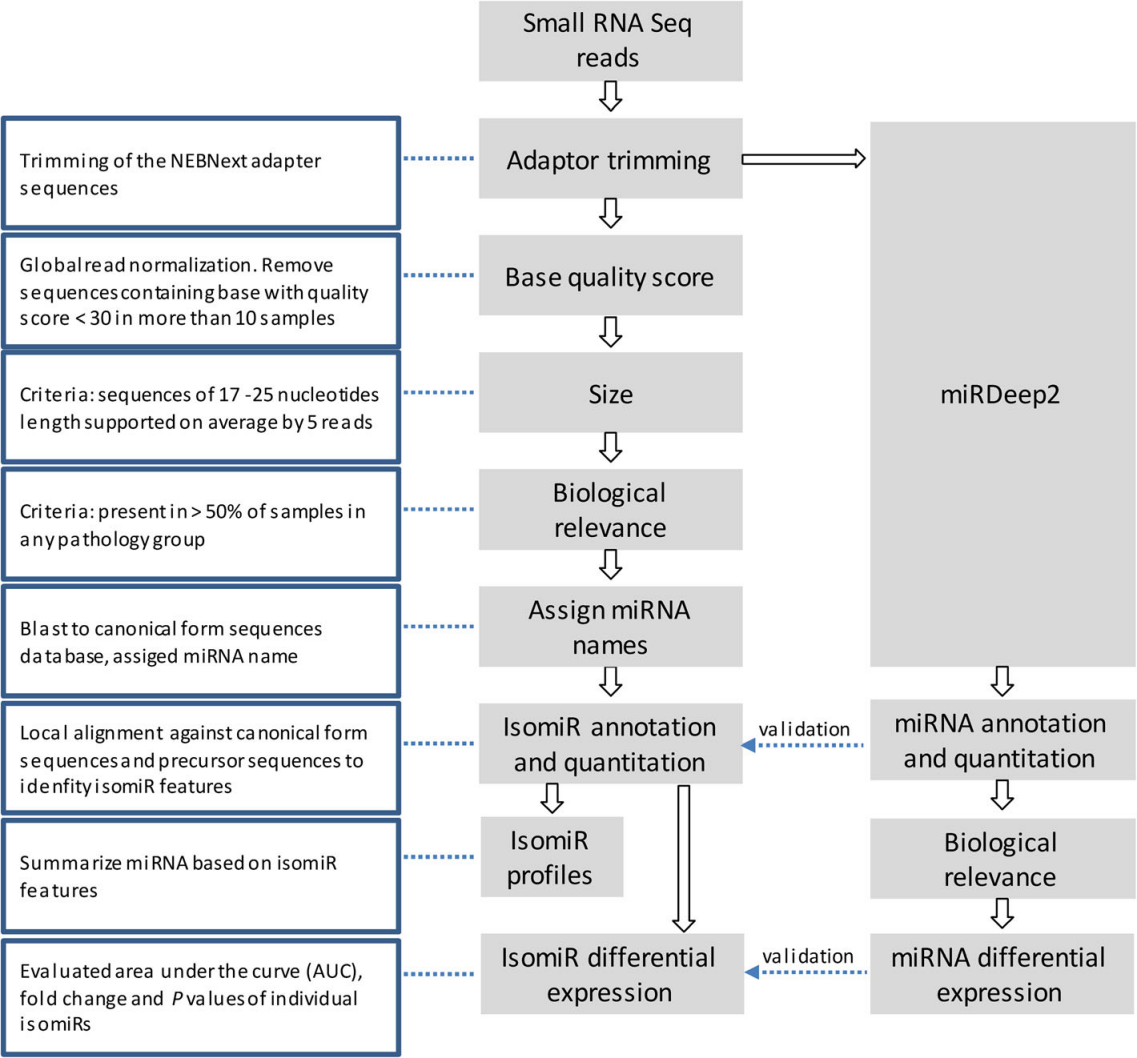
Schematic diagram of CASMIR workflow. miRDeep2 was used to validate annotation and differential expression based on the isomiR workflow

NEBNext adapter sequences were trimmed form the 50 bps Illumina HiSeq 2000 reads [20] (Additional file 1: Table S1). Small RNA reads were first scaled to total trimmed reads of the sample and further normalized across samples using a localized smoothing algorithm [21]. Following criteria were adopted: *1*) removal of sequence containing base(s) with quality score less than 30 presenting in more than 10 samples. This eliminates variants originated from sequencing artifacts. *2*) 17–25 nucleotides in length, which defines the typical sizes of miRNA; *3*) average read above 5; *4*) present in at least 50% samples of any pathology group (Fig. 2).

#### Assigning canonical form

Sequences were aligned to mature miRNA database version 21 (ftp://mirbase.org/pub/mirbase/21/) to identify their respective canonical sequences using our custom isomiR-BLAST alignment tool (https://github.com/jaredmevans/isomiR-BLAST). Alignment parameters included a word size of 13, gap open penalty of 5, gap extension penalty of 2, and required a forward strand match. Here, we adopted parameters consistent with the miRNA alignment in the miRDeep2 algorithm to facilitate subsequent comparisons with the miRDeep2 annotation. Instead of using short-read aligners such as BWA and Bowtie to interrogate the entire genome, custom blasting against targeted database can significantly reduce analysis runtime.

#### Local alignment to identify isomiR features

miRNA sequences were first compared to respective canonical forms based on Smith–Waterman algorithm of local alignment to identify mismatch and gap locations [22]. Five prime or 3′ additional forms were further aligned to precursor sequences to determine template or non-template form. Sequences were annotated with comprehensive isomiR features using the previously described classification. Scripts to implement this function are available at https://github.com/shengbing/IsoMIR-local-alignment-and-classification

### miRDeep2 workflow

In parallel, we ran miRDeep2, an established miRNA analysis pipeline which summarizes reads under mature miRNA names [20, 23]. Reads were scaled to total reads that can be aligned to miRNA and further normalized across samples using localized smoothing algorithm [21]. Two miRNA inclusion criteria were adopted: *1*) average reads above 10 and *2*) presence in at least 50% samples of any phenotypic group. Spearman r was used to evaluate correlation in reads between the two pipelines.

### Differential expression analysis

A sample size of 20 was based on detecting a minimum area under an receiver operating characteristic curve (AUC) of 0.75 above random classification (AUC = 0.5) between any two subgroups with 80% power assuming using a one-sided significance level of 5%. We used a Poisson regression model to estimate fold-change between groups with corresponding *P* values. A quasi-likelihood approach was used to adjust the Poisson regression model for over dispersion present in the data. AUC was estimated with test of significance based on the methods of DeLong, DeLong, and Clarke-Pearson [24]. Expression profiles of isomiR and miRNA, annotated by the isomiR workflow and miRdeep2, respectively, were ranked by AUCs for endpoints of colorectal cancer or advanced adenoma.

### Differentiation of isomiRs from canonical forms by qRT-PCR

To evaluate the advantage of targeting isomiRs as biomarkers, isomiRs and their canonical forms were tested by qRT-PCR in the *validation set*. Because qRT-PCR has limitations in differentiation of from highly similar canonical forms, custom assay designs and PCR condition optimization were required. For this process, we selected isomiRs for testing on the following considerations: *1*) isomiRs with AUC superior to respective canonical forms based on sequencing data, *2*) 3′ isomiRs, to allow specific qPCR assay design, and *3*) feasibility for the custom designed assays to differentiate the isomiR from canonical, as confirmed by empirical testing on synthetic RNA oligo (Additional file 2: Figure S1). For this third consideration, we adopted locked nucleic acid (LNA) platform with customization (Exiqon, Denmark). Details on oligo sequences and Exiqon assay numbers were provided (Additional file 3: Table S2). All qRT-PCR reactions were run on a LightCycler 480 System (Roche, Switzerland). Quantification cycle number (Cq) was analyzed using automatic baseline adjustment. AUCs were calculated using Graphpad Prism 6.0. All qRT-PCRs were performed by investigators who were blinded to samples’ clinical information.

## Results

### IsomiR profiling of colorectal epithelia

**Fig. 3:**
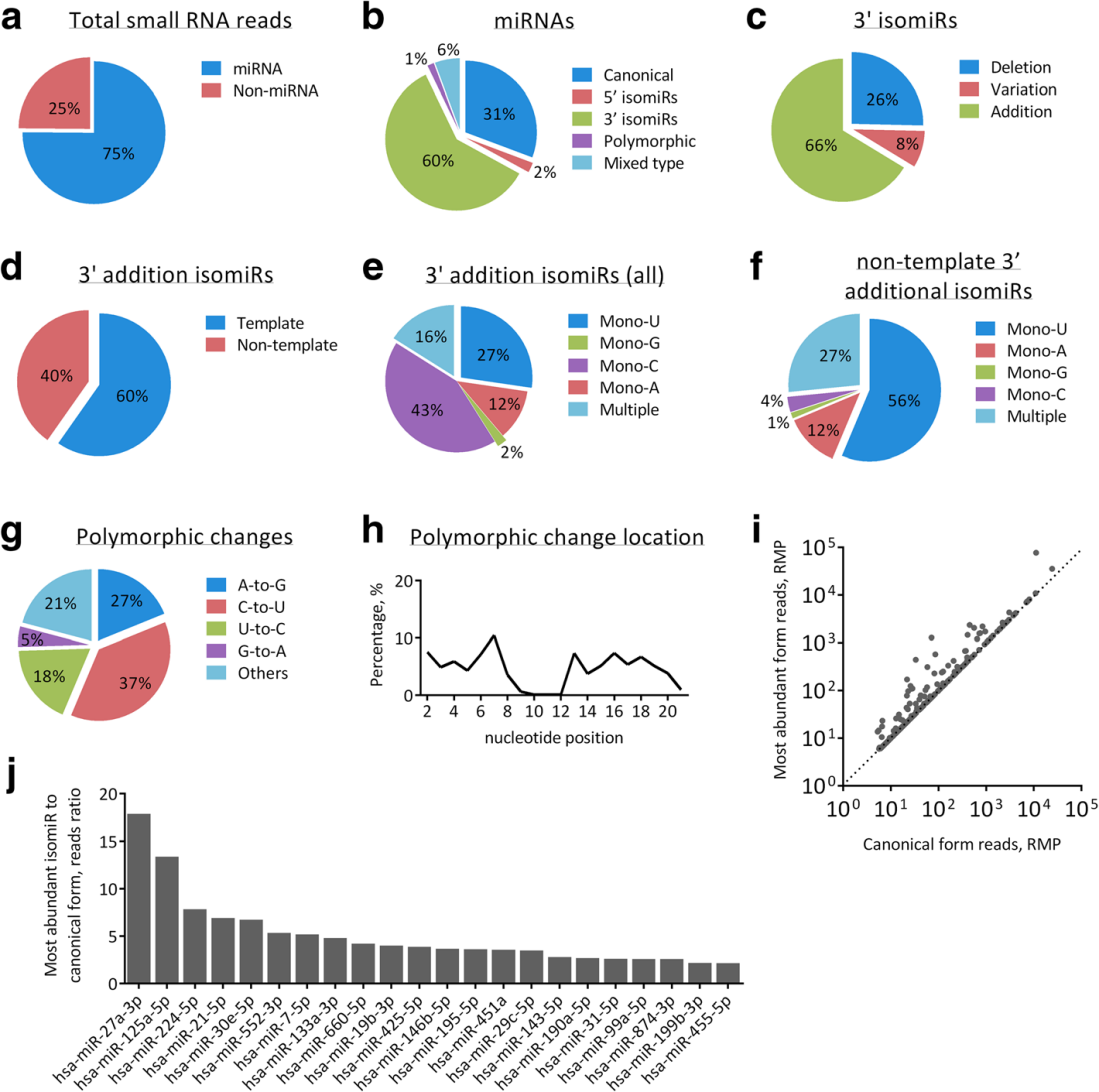
IsomiR profile of colorectal tissue. **a** miRNA reads among total mappable small RNA reads. **b** Canonical form and other isomiR among total miRNA reads. **c** Addition, deletion and variation forms among all 3′ isomiRs. **d** Template and non-template forms of 3′ addition form. **e** Additional nucleotide pattern of all 3′ addition form. **f** Additional nucleotide pattern of non-template 3′ addition form. **g** Polymorphic change nucleotide pattern. **h** Polymorphic change frequency based on nucleotide position as a percentage of total incidence. **i** Correlation in reads between the most abundant forms and canonical forms of each miRNA. Dots aligned on the diagonal line represent miRNAs whose canonical forms are also the most abundant forms. Otherwise, dots left to the diagonal line represent miRNAs with the most abundant forms being isomiRs. **j** miRNAs with highest ratios between the most abundant form reads to canonical form reads

Small RNA sequencing on the 81 colorectal tissue samples generated 317 million total raw reads with an average read of 3.9 million per sample. We have shown previously that a read depth of ~ 4 million allowed identification of 100% of miRNAs with above 50 reads and 97% of miRNAs above 15 reads detectable by a 24 million read depth [20]. No significant difference in average read count was found among disease groups. Among reads that met the filtering criteria (i.e. base quality score ≥ 30; 17–25 nucleotides in length; average read above 5; present in at least 50% samples of any group), there were 6568 unique sequences. Roughly 75% of the qualified reads were mapped to miRNA (Fig. 3a), comprising 2937 unique miRNA sequences. Based on all 81 samples, canonical forms account for 31% of total miRNA reads, while non-canonical isomiRs account for 69%. 3′ isomiR is the most predominant form of isomiR, contributing 60% of all isomiRs (Fig. 3b), and 66% of all 3′ isomiRs fell into the addition form (Fig. 3c). Sixty percent 3′ addition isomiRs are template forms, of which the additional nucleotides could be mapped to the parental precursor sequences (Fig. 3d). Based on nucleotide patterns, the addition of a single cytosine (C), uracil (U) and adenine (A) account for 43, 27 and 12%, respectively, of all 3′ addition isomiRs (Fig. 3e). Notably, miR-21-5p accounts for 89% of total 3′ addition C. IsomiR profiles based on global miRNA of pathology groups and miRNA arm features are shown in Additional file 4: Table S3 and Additional file 5: Table S4, respectively. 5′ isomiRs and polymorphic isomiRs are rare, accounting for only 2 and 1%, respectively, of total miRNA reads. The most frequent polymorphic change is C-to-U change (Fig. 3g). Polymorphic changes are almost absent in positions 9 to 12, and were distributed relatively evenly across the rest of the sequence (Fig. 3h). This pattern is consistent across pathology groups, and miRNA arm features (Additional file 6: Figure S2).

#### Non-template addition forms of isomiRs

To identify the addition forms that are generated by post-cleavage modification instead of alternative cleavage, we examined isomiRs with non-template 3′ addition. As their additional nucleotides did not match the precursor sequences, they could only be generated by post-cleavage modification. The addition of single U and A are the most common, accounting for 56 and 12% of all non-template 3′ addition isomiR (Fig. 3f). When analyze based on arm features, nearly 73% of non-template 3′ addition isomiR among 3p arm generated miRNA demonstrate 3′ addition of single U, compared to only 25% with 5p arm generated miRNA (Additional file 5: Table S4).

#### Most abundant isomiRs

Based on all 81 small RNA libraries, the most abundant species is often a non-canonical isomiR (Fig. 3i). With some miRNAs, non-canonical isomiRs could exceed the canonical form by as much as 18-fold (Fig. 3j). Among them, read count of the most abundant isomiRs of miR-27a-3p (3′ deletion C), miR-125a-5p (3′ deletion GA), miR-224-5p (3′ addition U), miR-21-5p (3′ addition C), and miR-30e-5p (3′ addition CU) are 18, 13, 8, 7, and 7-fold higher than their respective canonical forms.

#### IsomiR annotation validation by reads counts and clinical characteristics

**Fig. 4:**
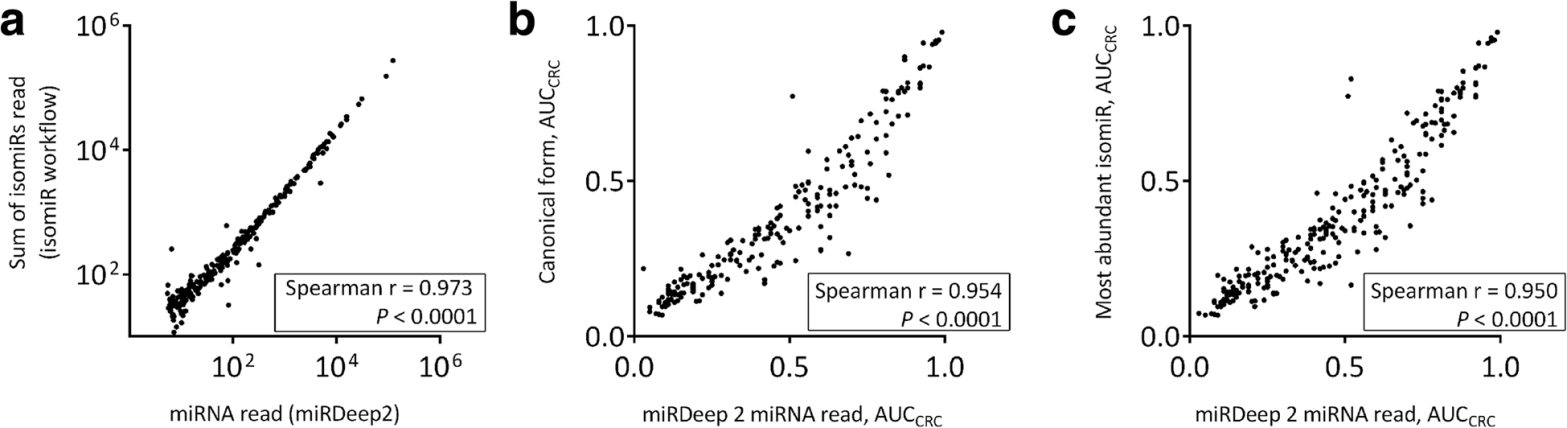
Validation of CASMIR annotation by miRDeep2 using read counts and AUC features. **a** IsomiR reads of each miRNA were summed and correlated with miRNA read quantified by miRDeep2. The two quantifications correlate significantly with spearman *r* = 0.973. Cancer AUCs generated by (**b**) canonical form reads, and **c** most abundant isomiR reads quantified by the isomiR pipeline correlate with AUCs generated by miRdeep2 reads with spearman *r* = 0.954 and 0.950, respectively, indicating the high consistency between the two workflows

To validate the isomiR annotation, isomiR reads of each miRNA were summed and correlated with miRNA read quantified by miRDeep2. Reads identified by the two workflows correlate with Spearman *r* = 0.973 with statistical significance (*P* < 0.0001, Fig. 4a), indicating that the sequence criteria defined in this study were efficient in covering most isomiRs. Users could make adjustment to the criteria according to the species interrogated. We also investigated the consistency of AUCs generated based on isomiR reads and miRDeep2 reads. AUCs generated by canonical form (Fig. 4b) or the most abundant isomiRs (Fig. 4c) correlate with AUCs generated by miRDeep2 reads with Spearman *r* = 0.954 and 0.950, respectively, at statistically significantly levels (both *P* < 0.0001).

### miRNA differential expression at isomiR level

**Fig. 5:**
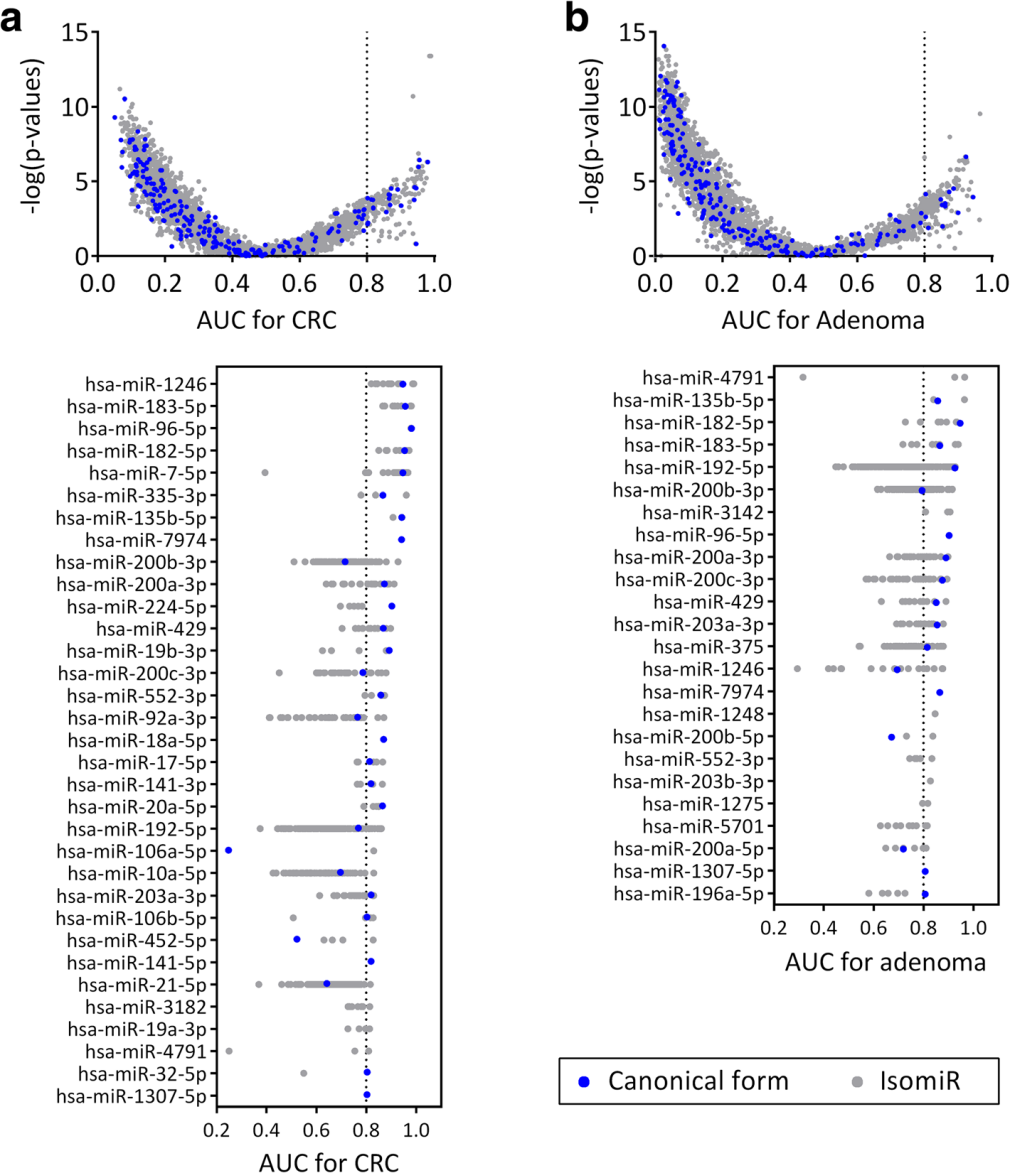
miRNA differential expression at isomiR level in colorectal neoplasia. Volcano plots of *P*-values against AUC demonstrated miRNA differential expression in (**a**) CRC and (**b**) advanced adenoma. Vertical dash lines indicate cutoff at AUC > 0.8 identifying those sequences with substantially unregulated expression. Lower panels indicate the AUC range of isomiRs among miRNAs with at least one isomiR with AUC > 0.8

We analyzed the 2937 unique sequences for differential expression between the normal and neoplastic groups. We defined downregulation as AUC < 0.2 and *P* < .01, and upregulation as AUC > 0.8 and *P* < .01 in differentiating neoplasm from normal. Among 2937 sequences, 631 and 1250 sequences were downregulated in CRC (Fig. 5a) and advanced adenoma (Fig. 5b), respectively. Meanwhile, only 144 and 131 sequences were upregulated in CRC and advanced adenoma (Additional files 7 and 8: Table S5 and S6), respectively. The predominant downregulation of global miRNA expression in neoplasia is consistent with previous reports [25]. A majority of the upregulated isomiRs belong to miRNA families implicated in tumorigenesis including miR-17-92, miR-200, and miR-183 families. Among them, 58 sequences are consistently upregulated in both CRC and advanced adenoma (Additional file 9: Table S7), including isomiRs of hsa-miR-135b-5p, − 182-5p, − 183-5p, − 192-5p, −200b-3p, − 96-5p, −200a-3p, −200c-3p, and − 429. IsomiRs of a particular miRNA demonstrate a wide variation in AUC. Importantly, the most discriminant forms are often specific non-canonical isomiRs instead of the canonical forms (Fig. 5a and b).

### Comparison with TCGA data

Using the Cancer Genome Atlas (TCGA) dataset, Telonis et al. [10] identified differentially expressed isomiRs in 32 cancer types. Their analysis focused only on template form isomiRs using an in-house analysis pipeline. Nevertheless, among miRNA most specific to colon and rectal cancers identified in TCGA, 66% (59 canonical and non-canonical isomiRs belonging to 23 miRNAs) could also be identified in our colorectal cancer samples (Additional file 10: Table S8); 57 of these 59 were consistently found to be over-expressed in CRC compared to normal epithelia. Since CASMIR is not limited to identifying template form isomiRs, Additional file 10: Table S8 shows comprehensive isomiRs of these 23 miRNAs identified by CASMIR in our dataset, including a total of 171 template isomiRs and 679 non-template form isomiRs.

### Targeting isomiRs by quantitative reverse-transcription PCR

**Fig. 6:**
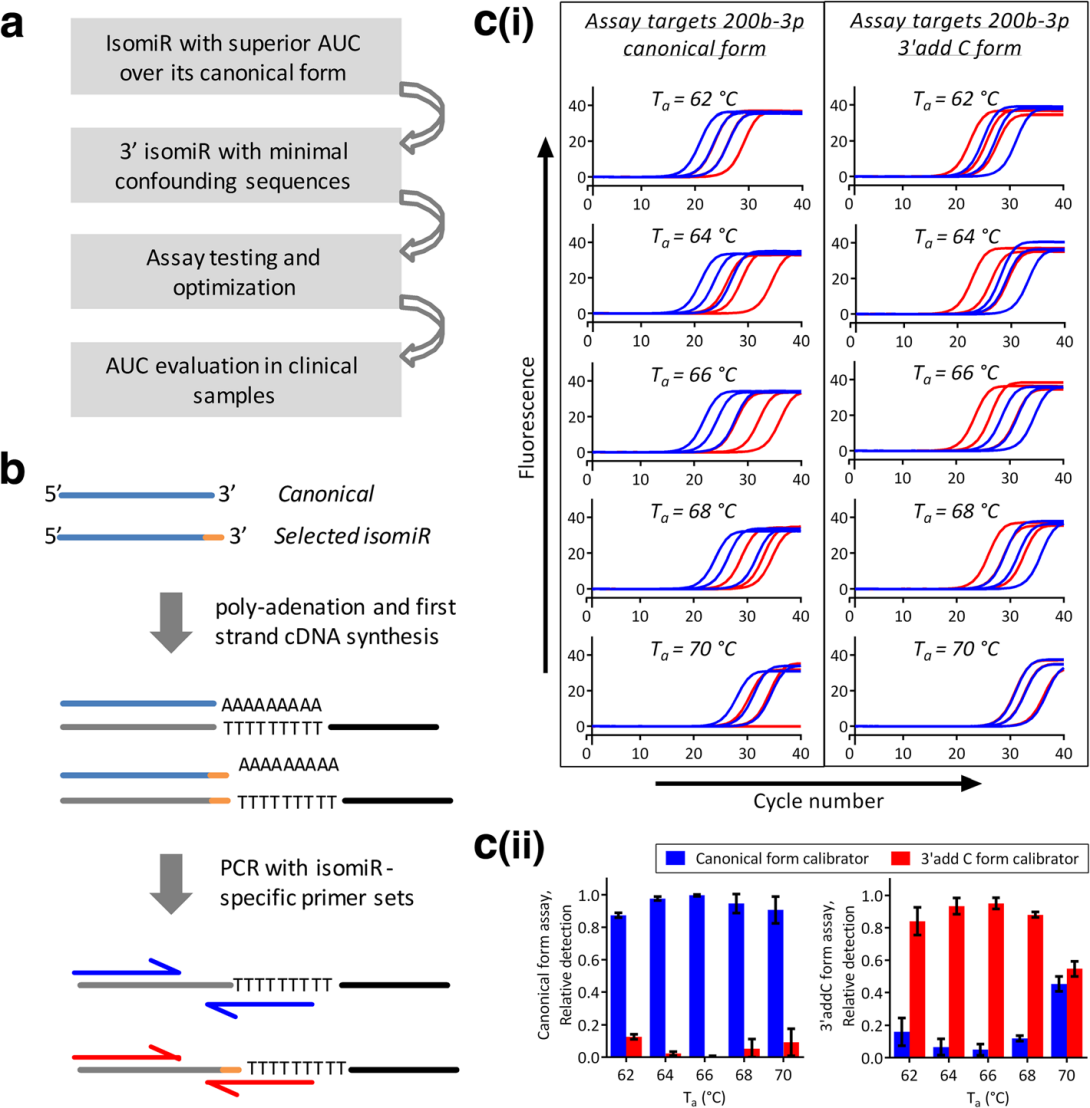
Differentiation of isomiRs from their canonical forms by qRT-PCR. **a** selection considerations. **b** Schematic diagram of assay design. Exiqon’s miRNA qRT-PCR assay involves polyadenylation at the 3′ end, rendering the assay high 3′ end specificity. Through optimizing qPCR conditions, they can differentiate 3′ isomiR from canonical forms. **c** Example on qPCR condition optimization: PCR annealing temperature (T_a_) testing for maximal differentiation between canonical form (hsa-miR-200b-3p) and its isomiR (3′ addition C form). **c i** Amplification of canonical form oligos (10^4^, 10^3^, 10^2^ copies; blue curves) and 3′ addition C form oligos (10^4^, 10^3^, 10^2^ copies; red curves) by assay targeting canonical form (left panel), and assay targeting 3′ addition C form (right panel), respectively, under various T_a_ (PCR profile refers to Additional file 2: Figure S1); **c ii** Summary of T_a_ testing, bar charts represent relative detection of canonical form oligos (red) and 3′ addition C form oligos (blue). Data represent mean ± s.d. of three data points based on the amplification of 10^4^, 10^3^, and 10^2^ copies of oligos

We selected nine 3′ isomiRs with superior AUCs compared to their canonical forms in discriminating CRC and/or advanced adenoma from controls (Additional file 3: Table S3). Pre-designed miRNA assays for canonical forms detected isomiRs in a non-specific manner. For instance, pre-designed hsa-miR-200a-3p assay primarily detect the canonical form but not its 3′ deletion U form (Additional file 11: Figure S3g); a pre-designed assay for hsa-miR-17-5p is more efficient in detecting 3′ addition U form using an annealing temperature (T_a_) above 64 °C (Additional file 11: Figure S3b). Upon optimization, we identified four isomiRs (hsa-miR-17-5p 3′ addition U, hsa-miR-21-5p 3′ addition C, hsa-miR-141-3p 3′ addition C, and hsa-miR-200b-3p 3′ addition C) which could be differentiate from their respective canonical forms through customized PCR conditions (Fig. 6c and Additional file 11: Figure S3b, c, and e). Differentiation of hsa-miR-21-5p 3′ addition C and hsa-miR-200b-3p 3′ addition C from their canonical forms were the most robust. In the *validation set*, all four isomiRs retained higher discrimination than their respective canonical forms based on triplicated experiments (Additional file 12: Table S9).

## Discussion

Our study aimed to address the current lack of consistency and comprehensiveness in annotating isomiRs in small RNA sequencing data. The CASMIR approach has two important features. First, it maintains isomiR reads as unique sequences to preserve mismatch features. While other tools summarize them under mature miRNA names, CASMIR characterizes mismatch features according to a structured classification to address the fact that each miRNA represents a diversity of sequences. As each sequence is unique, they can be used as identifiers to summarize data across large sample sets for statistical analysis.

Second, instead of limiting annotation to one or two isomiR features or pre-defined isomiR subsets, CASMIR identifies all isomiRs in small RNA sequencing data, facilitating summarization of global isomiRs in various contexts for analysis. In our *discovery set* consisting of 81 colorectal epithelial samples, we found that the 3′ addition is the most predominant isomiR form. Mono-uridylation is the most common 3′ end non-template modification and is more common in 3p-arm miRNAs. IsomiRs with polymorphic changes at the 5′ seed region are uncommon, accounting for less than 0.5% of all miRNA reads, indicating that many isomiRs may potentially affect the same downstream targets as their canonical sequences. Non-template form 5′ modifications appear to be rare.

A few factors could potentially affect the observed isomiR population. FFPE samples experience more extensive RNA degradation than fresh frozen samples. Previously, we showed that matched frozen and FFPE samples demonstrated a high consistency in global miRNA expression [26]. In this study, we observed a significant abundance of non-template addition isomiRs, which are very unlikely results of random degradation. Second, the ligation step in small RNA library preparation is known to bias against certain RNA-adapter secondary structures [27, 28]. Such bias is not dependent on terminal bases, therefore, might not affect the majority 5′ and 3′ isomiRs identification. We accessed data of independent samples from TCGA analyzed by an independent pipeline focused on template form isomiR and were able to observe a high reproducibility of the same isomiRs. Nevertheless, platform dependent bias in isomiR identification is not thoroughly addressed in this study and deserves further elucidation.

The biological significance of 3’end modifications has been nicely elucidated by others. Mainly, they modulate miRNA processing, stability, argonaute loading, and targeting effectiveness [4, 6, 29, 30]. We found that 3′ end mono-uridylation is much more abundant in miRNAs generated from 3p-arm than those from 5p-arm. This is consistent with in vitro findings that as Dicer prefers 2 nucleotides 3′ overhang in the precursor hairpin for processing [31], certain hairpins that has only one 3′ overhang nucleotide dependent on 3′ end mono-uridylation to gain the addition nucleotide for Dicer processing [30], resulting in a predominant mono-uridylation at the 3′ end of the 3p-arm generated miRNA.

Though their presence is well-established, isomiRs are often overlooked in functional studies due to the fact that the predominant modifications at 3′ end are not likely to alter the seed region at 5′ end [32]. However, as candidate biomarkers for detection, defining miRNA at the isomiR level could be of critical importance to optimizing diagnostic discrimination. The current miRBase lists one “mature sequence” per miRNA, which is usually the sequence with the highest coverage registered in the database according to species. Based on our findings, specific isomiRs are more abundant than the canonical forms; some exceed their canonical form by more than 10-fold. It is likely isomiR expression is dynamic. Tissues or cell-type isomiR profile could inform which isomiR is the most relevant. In the context of biomarker, findings from isomiR differential expression analysis were consistent with previous reports on upregulated miRNAs in CRC, including on hsa-miR-135b [19], − 21-5p [33], 92a-3p [33], − 7-5p [34], − 17-5p [35], − 182-5p [36, 37], − 183-5p [38], − 1246 (or U2 small nuclear RNA fragments) [39] and miR-200 family [40]. Our analyses at the isomiR level further identified a rich and previously unseen layer of candidate sequences. We found that for a majority of these miRNAs, specific isomiRs are often more discriminant than their canonical forms. For instance, hsa-miR-21-5p, a widely studied miRNA upregulated in CRC, was more abundant and discriminant in its 3′ addition C form than in canonical form in colorectal epithelia. Its superior performance as a biomarker was validated in an independent patient cohort using qRT-PCR. As many upregulated miRNAs in cancer have been implicated as biomarkers in distant media, it may be advantageous to target isomiRs with superior operating characteristics. Further applied clinical and translational research targeting such isomiR marker candidates is clearly indicated.

The majority 3′ end modification imposes challenge to their detection. Existing miRNA qRT-PCR assays, which are limited to proprietary methods, use either poly-adenylation or stem-loop reverse transcription [41]. Both methods generate elongated complementary DNA (cDNA) template from miRNA 3’end, rendering relatively higher specificity towards 3′ end sequence. Therefore, pre-designed assays intended for canonical forms are not specific to 3′ isomiRs. Others have also described the challenges of using qPCR based methods to detect isomiRs [42, 43]. We described an isomiR selection process and assay customization process to overcome some of these challenges. However, given the complexity of this process and its limitation in throughput and specificity, we suggest that a more practical approach for interrogation of a panel or the global isomiR profile would be through NGS and potentially by including molecular barcodes to improve specificity.

## Conclusions

IsomiRs are biologically relevant variants that significantly increase the existing miRNA repertoire focused on canonical forms. Studying miRNAs at the isomiR level, which has received little emphasis historically, could bring new biological insights and inform development of miRNA-based diagnostic assays. CASMIR is a stepwise approach for annotating and summarizing isomiRs in small RNA sequencing data. Depending on the intended application, CASMIR can be performed as a standalone workflow or alongside a standard miRNA pipeline such as miRDeep2 to support isomiR subtyping. We maintain CASMIR as an unpackaged tool to allow customized applications. CASMIR has potential to facilitate the study of miRNA through its unbiased and comprehensive identification of isomiRs.

## Additional files

Additional file 1: **Table S1.** Total reads and trimmed read pre sample. (XLSX 12 kb)

Additional file 2: **Figure S1.** PCR condition optimization for the differentiation of isomiR from canonical form. (DOCX 163 kb)

Additional file 3: **Table S2.** Selected non-canonical form isomiRs with superior AUCs compared to their respective canonical forms. (XLSX 11 kb)

Additional file 4: **Table S3.** Comprehensive isomiR profile based on pathology groups. (XLSX 15 kb)

Additional file 5: **Table S4.** Comprehensive isomiR profile based on miRNA arm features. (XLSX 13 kb)

Additional file 6: **Figure S2.** Polymorphic change pattern. (DOCX 154 kb)

Additional file 7: **Table S5.** Most upregulated isomiRs in colorectal cancer. (XLSX 80 kb)

Additional file 8: **Table S6.** Most upregulated isomiRs in advanced adenoma. (XLSX 107 kb)

Additional file 9: **Table S7.** Commonly upregulated isomiRs in colorectal cancer and advanced adenoma. (XLSX 40 kb)

Additional file 10: **Table S8.** Reproducibility of isomiRs identified in TCGA dataset using an independent pipeline and in our dataset using CASMIR. (XLSX 111 kb)

Additional file 11: **Figure S3.** Pre-amplification step annealing temperature optimization. (DOCX 704 kb)

Additional file 12: **Table S9.** AUCs in differentiating colorectal neoplasm from normal in *Validation set* using isomiRs. (XLSX 9 kb)

## Abbreviations

AUC: Area under a receiver operating characteristic curve
CASMIR: Comprehensive approach to sequence-oriented isomiR annotation
CRC: Colorectal cancer
FFPE: Formalin-fixed paraffin-embedded
miRNA: microRNA
NGS: Next generation sequencing
qRT-PCR: Quantitative reverse-transcription polymerase chain reaction
TCGA: the Cancer Genome Atlas
