# Supplementary material for: A Comprehensive Approach to Sequence-oriented IsomiR annotation (CASMIR): demonstration with IsomiR profiling in colorectal neoplasia
Source: BMC Genomics. 2018 May 25;19:401. doi: 10.1186/s12864-018-4794-7 (PMC5970459; doi:10.1186/s12864-018-4794-7)
Supplement: Supplementary file 2 — Figure S1. PCR condition optimization for the differentiation of isomiR from canonical form. (DOCX 163 kb) [file 12864_2018_4794_MOESM2_ESM.docx]

**Fig. S1.** PCR condition optimization. Non-canonical form isomiR often differs from its canonical form by a single nucleotide. They could be difficult to differentiate by PCR. To improve assay specificity from the manufacturer’s standard protocol as shown in (*A*), a pre-amplification step of 10 cycles with high annealing temperature (T_a_) prior to signal acquisition cycles was used to enrich specific target (*B*). Pre-amplification T_a_ was determined by the detection of both selected non-canonical form and canonical form calibrators (10^4^, 10^3^, 10^2^ copies), T_a_ that can best differentiate selected non-canonical form from canonical form was adopted in subsequent testing on clinical samples.
