# Supplementary material for: A Comprehensive Approach to Sequence-oriented IsomiR annotation (CASMIR): demonstration with IsomiR profiling in colorectal neoplasia
Source: BMC Genomics. 2018 May 25;19:401. doi: 10.1186/s12864-018-4794-7 (PMC5970459; doi:10.1186/s12864-018-4794-7)
Supplement: Supplementary file 6 — Figure S2. Polymorphic change pattern. (DOCX 154 kb) [file 12864_2018_4794_MOESM6_ESM.docx]

**Fig. S2** Polymorphic change frequency pattern. Polymorphic change frequency based on nucleotide position in (**a**) normal colorectal epithelia, (**b**) adenoma (**c**) CRC, (**d**) 3p arm miRNAs among all samples, and (**e**) 5p arm miRNA among all samples. y-axis indicates change at a certain nucleotide position as a percentage of total incidence of polymorphic change.
