# Supplementary material for: A Comprehensive Approach to Sequence-oriented IsomiR annotation (CASMIR): demonstration with IsomiR profiling in colorectal neoplasia
Source: BMC Genomics. 2018 May 25;19:401. doi: 10.1186/s12864-018-4794-7 (PMC5970459; doi:10.1186/s12864-018-4794-7)
Supplement: Supplementary file 11 — Figure S3. Pre-amplification step annealing temperature optimization. (DOCX 704 kb) [file 12864_2018_4794_MOESM11_ESM.docx]

**Fig. S3** Pre-amplification step annealing temperature (T_a_) optimization. Optimizations of assays for (**a**) hsa-miR-7-5p and its 3'addition U form, (**b**) hsa-miR-17-5p and its 3'addition U form assay, (**c**) hsa-miR-21-5p and its 3'addition C form assay, (**d**) hsa-miR-135b-5p and its 3'deletion A form assay, (**e**) hsa-miR-141-3p and its 3'addition C form assay, (**f**) hsa-miR-196b-5p and its 3'deletion G form assay, (**g**) hsa-miR-200a-3p and its 3'addition U form assay, and (**h**) hsa-miR-335-3p and its 3'deletion C form assay. Bar charts represent relative detection of canonical form calibrators (red) and the selected non-canonical form calibrator (blue) by their respective assays. Cross-reactivity could be minimized by optimizing T_a_. Data represent mean ± s.d. of three data points based on the amplification of 10^4^, 10^3^, and 10^2^ copies of calibrators, respectively.
